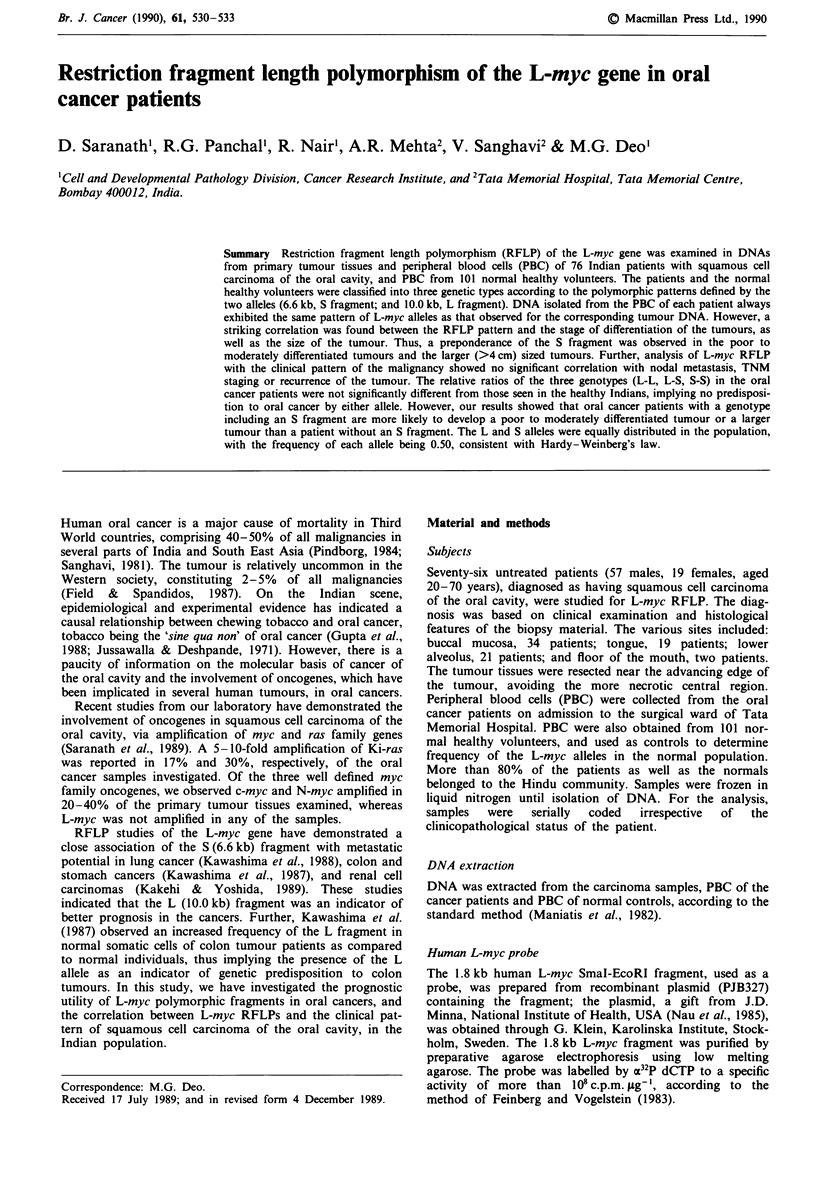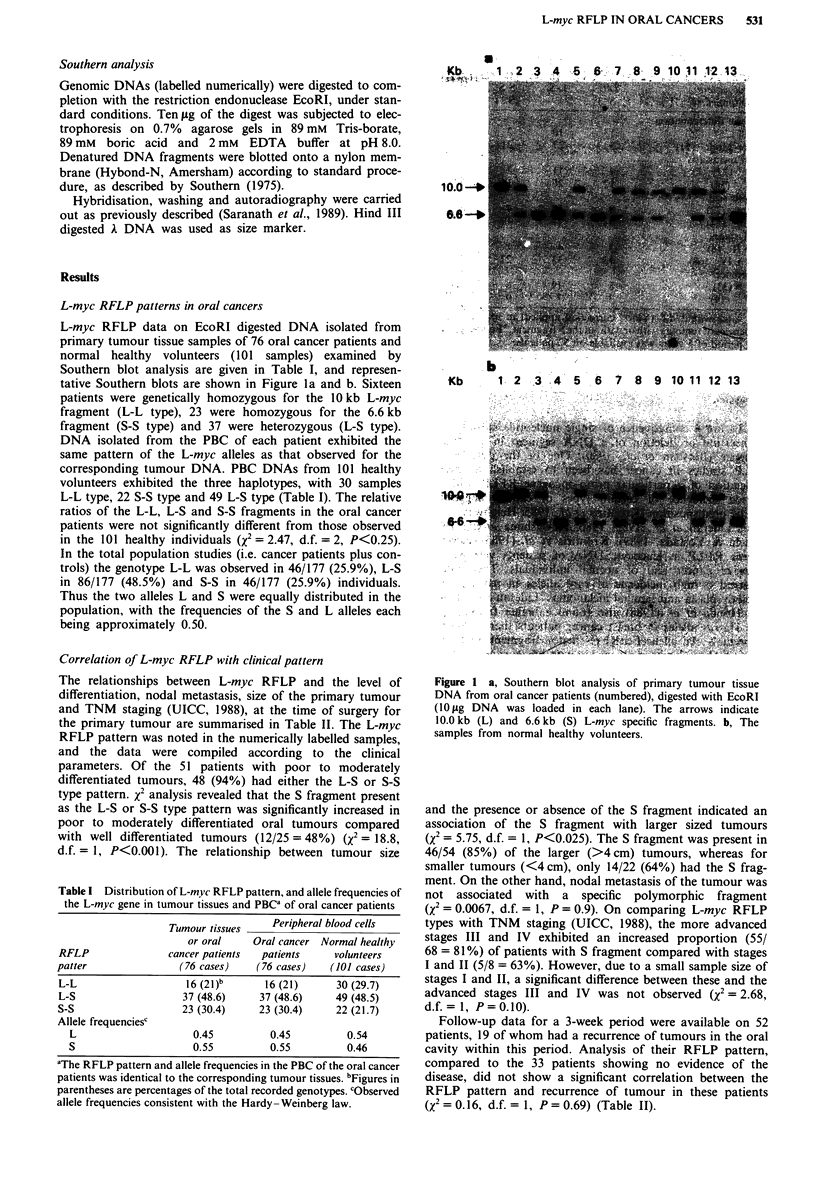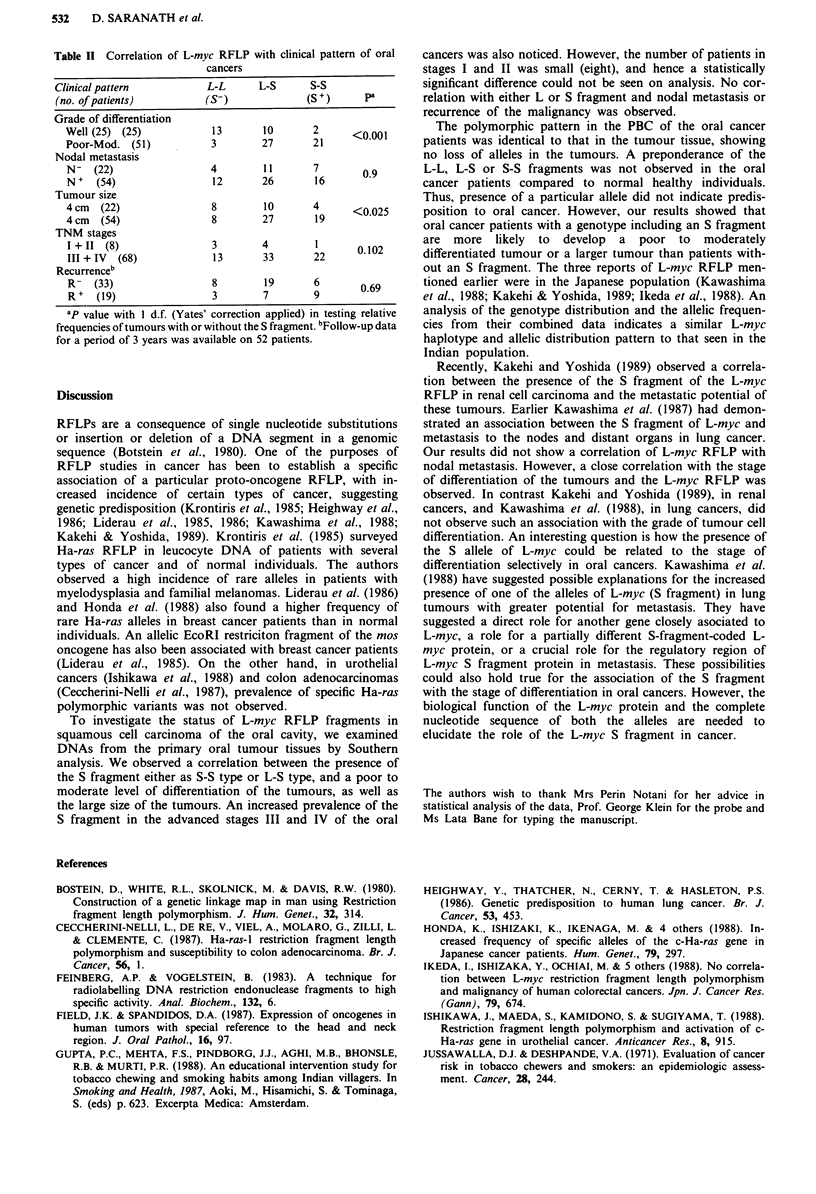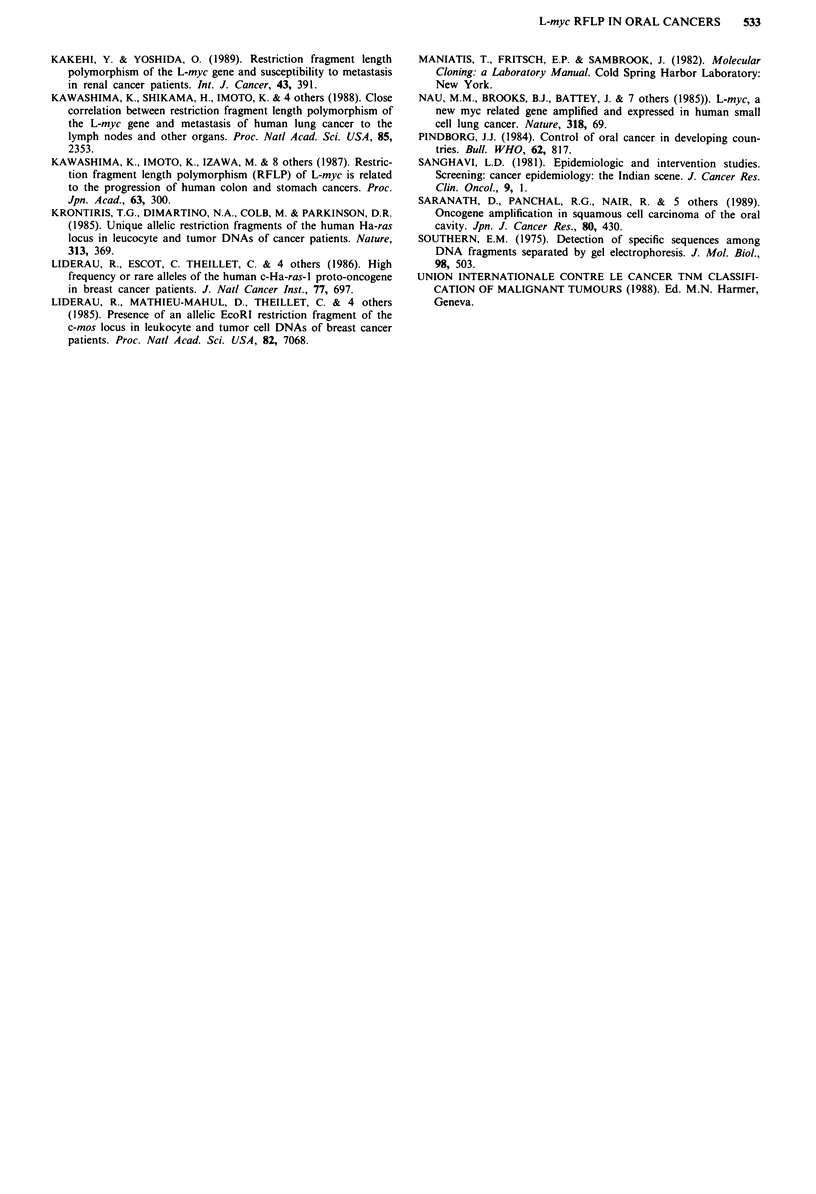# Restriction fragment length polymorphism of the L-myc gene in oral cancer patients.

**DOI:** 10.1038/bjc.1990.119

**Published:** 1990-04

**Authors:** D. Saranath, R. G. Panchal, R. Nair, A. R. Mehta, V. Sanghavi, M. G. Deo

**Affiliations:** Cell and Developmental Pathology Division, Cancer Research Institute, Bombay, India.

## Abstract

**Images:**


Br. J. Cancer (1990), 61, 530-533 © Macmillan Press Ltd., 1990

### Restriction fragment length polymorphism of the L-*myc* gene in oral cancer patients

D. Saranath[1], R.G. Panchal[1], R. Nair[1], A.R. Mehta[2], V. Sanghavi[2] & M.G. Deo[1]

[1]*Cell and Developmental Pathology Division, Cancer Research Institute, and* [2]*Tata Memorial Hospital, Tata Memorial Centre, Bombay 400012, India.*

**Summary** Restriction fragment length polymorphism (RFLP) of the L-*myc* gene was examined in DNAs from primary tumour tissues and peripheral blood cells (PBC) of 76 Indian patients with squamous cell carcinoma of the oral cavity, and PBC from 101 normal healthy volunteers. The patients and the normal healthy volunteers were classified into three genetic types according to the polymorphic patterns defined by the two alleles (6.6 kb, S fragment; and 10.0 kb, L fragment). DNA isolated from the PBC of each patient always exhibited the same pattern of L-*myc* alleles as that observed for the corresponding tumour DNA. However, a striking correlation was found between the RFLP pattern and the stage of differentiation of the tumours, as well as the size of the tumour. Thus, a preponderance of the S fragment was observed in the poor to moderately differentiated tumours and the larger (>4 cm) sized tumours. Further, analysis of L-*myc* RFLP with the clinical pattern of the malignancy showed no significant correlation with nodal metastasis, TNM staging or recurrence of the tumour. The relative ratios of the three genotypes (L-L, L-S, S-S) in the oral cancer patients were not significantly different from those seen in the healthy Indians, implying no predisposition to oral cancer by either allele. However, our results showed that oral cancer patients with a genotype including an S fragment are more likely to develop a poor to moderately differentiated tumour or a larger tumour than a patient without an S fragment. The L and S alleles were equally distributed in the population, with the frequency of each allele being 0.50, consistent with Hardy-Weinberg's law.

Human oral cancer is a major cause of mortality in Third World countries, comprising 40-50% of all malignancies in several parts of India and South East Asia (Pindborg, 1984; Sanghavi, 1981). The tumour is relatively uncommon in the Western society, constituting 2-5% of all malignancies (Field & Spandidos, 1987). On the Indian scene, epidemiological and experimental evidence has indicated a causal relationship between chewing tobacco and oral cancer, tobacco being the '*sine qua non*' of oral cancer (Gupta *et al.*, 1988; Jussawalla & Deshpande, 1971). However, there is a paucity of information on the molecular basis of cancer of the oral cavity and the involvement of oncogenes, which have been implicated in several human tumours, in oral cancers.

Recent studies from our laboratory have demonstrated the involvement of oncogenes in squamous cell carcinoma of the oral cavity, via amplification of *myc* and *ras* family genes (Saranath *et al.*, 1989). A 5-10-fold amplification of Ki-*ras* was reported in 17% and 30%, respectively, of the oral cancer samples investigated. Of the three well defined *myc* family oncogenes, we observed c-*myc* and N-*myc* amplified in 20-40% of the primary tumour tissues examined, whereas L-*myc* was not amplified in any of the samples.

RFLP studies of the L-*myc* gene have demonstrated a close association of the S (6.6 kb) fragment with metastatic potential in lung cancer (Kawashima *et al.*, 1988), colon and stomach cancers (Kawashima *et al.*, 1987), and renal cell carcinomas (Kakehi & Yoshida, 1989). These studies indicated that the L (10.0 kb) fragment was an indicator of better prognosis in the cancers. Further, Kawashima *et al.* (1987) observed an increased frequency of the L fragment in normal somatic cells of colon tumour patients as compared to normal individuals, thus implying the presence of the L allele as an indicator of genetic predisposition to colon tumours. In this study, we have investigated the prognostic utility of L-*myc* polymorphic fragments in oral cancers, and the correlation between L-*myc* RFLPs and the clinical pattern of squamous cell carcinoma of the oral cavity, in the Indian population.

Correspondence: M.G. Deo.
Received 17 July 1989; and in revised form 4 December 1989.

### Material and methods

### Subjects

Seventy-six untreated patients (57 males, 19 females, aged 20-70 years), diagnosed as having squamous cell carcinoma of the oral cavity, were studied for L-*myc* RFLP. The diagnosis was based on clinical examination and histological features of the biopsy material. The various sites included: buccal mucosa, 34 patients; tongue, 19 patients; lower alveolus, 21 patients; and floor of the mouth, two patients. The tumour tissues were resected near the advancing edge of the tumour, avoiding the more necrotic central region. Peripheral blood cells (PBC) were collected from the oral cancer patients on admission to the surgical ward of Tata Memorial Hospital. PBC were also obtained from 101 normal healthy volunteers, and used as controls to determine frequency of the L-*myc* alleles in the normal population. More than 80% of the patients as well as the normals belonged to the Hindu community. Samples were frozen in liquid nitrogen until isolation of DNA. For the analysis, samples were serially coded irrespective of the clinicopathological status of the patient.

### DNA extraction

DNA was extracted from the carcinoma samples, PBC of the cancer patients and PBC of normal controls, according to the standard method (Maniatis *et al.*, 1982).

### Human L-myc probe

The 1.8 kb human L-*myc* SmaI-EcoRI fragment, used as a probe, was prepared from recombinant plasmid (PJB327) containing the fragment; the plasmid, a gift from J.D. Minna, National Institute of Health, USA (Nau *et al.*, 1985), was obtained through G. Klein, Karolinska Institute, Stockholm, Sweden. The 1.8 kb L-*myc* fragment was purified by preparative agarose electrophoresis using low melting agarose. The probe was labelled by $\alpha^{32}P$ dCTP to a specific activity of more than $10^8$ c.p.m. $\mu g^{-1}$, according to the method of Feinberg and Vogelstein (1983).

*Southern analysis*

Genomic DNAs (labelled numerically) were digested to completion with the restriction endonuclease EcoRI, under standard conditions. Ten μg of the digest was subjected to electrophoresis on 0.7% agarose gels in 89 mM Tris-borate, 89 mM boric acid and 2 mM EDTA buffer at pH 8.0. Denatured DNA fragments were blotted onto a nylon membrane (Hybond-N, Amersham) according to standard procedure, as described by Southern (1975).

Hybridisation, washing and autoradiography were carried out as previously described (Saranath *et al.*, 1989). Hind III digested λ DNA was used as size marker.

### Results

*L-myc RFLP patterns in oral cancers*

L-*myc* RFLP data on EcoRI digested DNA isolated from primary tumour tissue samples of 76 oral cancer patients and normal healthy volunteers (101 samples) examined by Southern blot analysis are given in Table I, and representative Southern blots are shown in Figure 1a and b. Sixteen patients were genetically homozygous for the 10 kb L-*myc* fragment (L-L type), 23 were homozygous for the 6.6 kb fragment (S-S type) and 37 were heterozygous (L-S type). DNA isolated from the PBC of each patient exhibited the same pattern of the L-*myc* alleles as that observed for the corresponding tumour DNA. PBC DNAs from 101 healthy volunteers exhibited the three haplotypes, with 30 samples L-L type, 22 S-S type and 49 L-S type (Table I). The relative ratios of the L-L, L-S and S-S fragments in the oral cancer patients were not significantly different from those observed in the 101 healthy individuals ($\chi^2 = 2.47$, d.f. = 2, $P<0.25$). In the total population studies (i.e. cancer patients plus controls) the genotype L-L was observed in 46/177 (25.9%), L-S in 86/177 (48.5%) and S-S in 46/177 (25.9%) individuals. Thus the two alleles L and S were equally distributed in the population, with the frequencies of the S and L alleles each being approximately 0.50.

*Correlation of L-myc RFLP with clinical pattern*

The relationships between L-*myc* RFLP and the level of differentiation, nodal metastasis, size of the primary tumour and TNM staging (UICC, 1988), at the time of surgery for the primary tumour are summarised in Table II. The L-*myc* RFLP pattern was noted in the numerically labelled samples, and the data were compiled according to the clinical parameters. Of the 51 patients with poor to moderately differentiated tumours, 48 (94%) had either the L-S or S-S type pattern. $\chi^2$ analysis revealed that the S fragment present as the L-S or S-S type pattern was significantly increased in poor to moderately differentiated oral tumours compared with well differentiated tumours (12/25 = 48%) ($\chi^2 = 18.8$, d.f. = 1, $P<0.001$). The relationship between tumour size

**Table I** Distribution of L-*myc* RFLP pattern, and allele frequencies of the L-*myc* gene in tumour tissues and PBC[a] of oral cancer patients

| *RFLP patter* | *Tumour tissues or oral cancer patients (76 cases)* | *Peripheral blood cells* | |
|---|---|---|---|
| | | *Oral cancer patients (76 cases)* | *Normal healthy volunteers (101 cases)* |
| L-L | 16 (21)[b] | 16 (21) | 30 (29.7) |
| L-S | 37 (48.6) | 37 (48.6) | 49 (48.5) |
| S-S | 23 (30.4) | 23 (30.4) | 22 (21.7) |
| Allele frequencies[c] | | | |
| L | 0.45 | 0.45 | 0.54 |
| S | 0.55 | 0.55 | 0.46 |

[a]The RFLP pattern and allele frequencies in the PBC of the oral cancer patients was identical to the corresponding tumour tissues. [b]Figures in parentheses are percentages of the total recorded genotypes. [c]Observed allele frequencies consistent with the Hardy–Weinberg law.

**Figure 1** **a**, Southern blot analysis of primary tumour tissue DNA from oral cancer patients (numbered), digested with EcoRI (10 μg DNA was loaded in each lane). The arrows indicate 10.0 kb (L) and 6.6 kb (S) L-*myc* specific fragments. **b**, The samples from normal healthy volunteers.

and the presence or absence of the S fragment indicated an association of the S fragment with larger sized tumours ($\chi^2 = 5.75$, d.f. = 1, $P<0.025$). The S fragment was present in 46/54 (85%) of the larger (>4 cm) tumours, whereas for smaller tumours (<4 cm), only 14/22 (64%) had the S fragment. On the other hand, nodal metastasis of the tumour was not associated with a specific polymorphic fragment ($\chi^2 = 0.0067$, d.f. = 1, $P = 0.9$). On comparing L-*myc* RFLP types with TNM staging (UICC, 1988), the more advanced stages III and IV exhibited an increased proportion (55/68 = 81%) of patients with S fragment compared with stages I and II (5/8 = 63%). However, due to a small sample size of stages I and II, a significant difference between these and the advanced stages III and IV was not observed ($\chi^2 = 2.68$, d.f. = 1, $P = 0.10$).

Follow-up data for a 3-week period were available on 52 patients, 19 of whom had a recurrence of tumours in the oral cavity within this period. Analysis of their RFLP pattern, compared to the 33 patients showing no evidence of the disease, did not show a significant correlation between the RFLP pattern and recurrence of tumour in these patients ($\chi^2 = 0.16$, d.f. = 1, $P = 0.69$) (Table II).

**Table II** Correlation of L-*myc* RFLP with clinical pattern of oral cancers

| *Clinical pattern (no. of patients)* | *L-L ($S^-$)* | L-S | S-S ($S^+$) | $P^a$ |
|---|---|---|---|---|
| Grade of differentiation | | | | |
| Well (25) (25) | 13 | 10 | 2 | <0.001 |
| Poor-Mod. (51) | 3 | 27 | 21 | |
| Nodal metastasis | | | | |
| $N^-$ (22) | 4 | 11 | 7 | 0.9 |
| $N^+$ (54) | 12 | 26 | 16 | |
| Tumour size | | | | |
| 4 cm (22) | 8 | 10 | 4 | <0.025 |
| 4 cm (54) | 8 | 27 | 19 | |
| TNM stages | | | | |
| I + II (8) | 3 | 4 | 1 | 0.102 |
| III + IV (68) | 13 | 33 | 22 | |
| Recurrence[b] | | | | |
| $R^-$ (33) | 8 | 19 | 6 | 0.69 |
| $R^+$ (19) | 3 | 7 | 9 | |

[a]*P* value with 1 d.f. (Yates' correction applied) in testing relative frequencies of tumours with or without the S fragment. [b]Follow-up data for a period of 3 years was available on 52 patients.

### Discussion

RFLPs are a consequence of single nucleotide substitutions or insertion or deletion of a DNA segment in a genomic sequence (Botstein *et al.*, 1980). One of the purposes of RFLP studies in cancer has been to establish a specific association of a particular proto-oncogene RFLP, with increased incidence of certain types of cancer, suggesting genetic predisposition (Krontiris *et al.*, 1985; Heighway *et al.*, 1986; Liderau *et al.*, 1985, 1986; Kawashima *et al.*, 1988; Kakehi & Yoshida, 1989). Krontiris *et al.* (1985) surveyed Ha-*ras* RFLP in leucocyte DNA of patients with several types of cancer and of normal individuals. The authors observed a high incidence of rare alleles in patients with myelodysplasia and familial melanomas. Liderau *et al.* (1986) and Honda *et al.* (1988) also found a higher frequency of rare Ha-*ras* alleles in breast cancer patients than in normal individuals. An allelic EcoRI restriciton fragment of the *mos* oncogene has also been associated with breast cancer patients (Liderau *et al.*, 1985). On the other hand, in urothelial cancers (Ishikawa *et al.*, 1988) and colon adenocarcinomas (Ceccherini-Nelli *et al.*, 1987), prevalence of specific Ha-*ras* polymorphic variants was not observed.

To investigate the status of L-*myc* RFLP fragments in squamous cell carcinoma of the oral cavity, we examined DNAs from the primary oral tumour tissues by Southern analysis. We observed a correlation between the presence of the S fragment either as S-S type or L-S type, and a poor to moderate level of differentiation of the tumours, as well as the large size of the tumours. An increased prevalence of the S fragment in the advanced stages III and IV of the oral cancers was also noticed. However, the number of patients in stages I and II was small (eight), and hence a statistically significant difference could not be seen on analysis. No correlation with either L or S fragment and nodal metastasis or recurrence of the malignancy was observed.

The polymorphic pattern in the PBC of the oral cancer patients was identical to that in the tumour tissue, showing no loss of alleles in the tumours. A preponderance of the L-L, L-S or S-S fragments was not observed in the oral cancer patients compared to normal healthy individuals. Thus, presence of a particular allele did not indicate predisposition to oral cancer. However, our results showed that oral cancer patients with a genotype including an S fragment are more likely to develop a poor to moderately differentiated tumour or a larger tumour than patients without an S fragment. The three reports of L-*myc* RFLP mentioned earlier were in the Japanese population (Kawashima *et al.*, 1988; Kakehi & Yoshida, 1989; Ikeda *et al.*, 1988). An analysis of the genotype distribution and the allelic frequencies from their combined data indicates a similar L-*myc* haplotype and allelic distribution pattern to that seen in the Indian population.

Recently, Kakehi and Yoshida (1989) observed a correlation between the presence of the S fragment of the L-*myc* RFLP in renal cell carcinoma and the metastatic potential of these tumours. Earlier Kawashima *et al.* (1987) had demonstrated an association between the S fragment of L-*myc* and metastasis to the nodes and distant organs in lung cancer. Our results did not show a correlation of L-*myc* RFLP with nodal metastasis. However, a close correlation with the stage of differentiation of the tumours and the L-*myc* RFLP was observed. In contrast Kakehi and Yoshida (1989), in renal cancers, and Kawashima *et al.* (1988), in lung cancers, did not observe such an association with the grade of tumour cell differentiation. An interesting question is how the presence of the S allele of L-*myc* could be related to the stage of differentiation selectively in oral cancers. Kawashima *et al.* (1988) have suggested possible explanations for the increased presence of one of the alleles of L-*myc* (S fragment) in lung tumours with greater potential for metastasis. They have suggested a direct role for another gene closely asociated to L-*myc*, a role for a partially different S-fragment-coded L-*myc* protein, or a crucial role for the regulatory region of L-*myc* S fragment protein in metastasis. These possibilities could also hold true for the association of the S fragment with the stage of differentiation in oral cancers. However, the biological function of the L-*myc* protein and the complete nucleotide sequence of both the alleles are needed to elucidate the role of the L-*myc* S fragment in cancer.

The authors wish to thank Mrs Perin Notani for her advice in statistical analysis of the data, Prof. George Klein for the probe and Ms Lata Bane for typing the manuscript.

### References

BOSTEIN, D., WHITE, R.L., SKOLNICK, M. & DAVIS, R.W. (1980). Construction of a genetic linkage map in man using Restriction fragment length polymorphism. *J. Hum. Genet.*, **32**, 314.

CECCHERINI-NELLI, L., DE RE, V., VIEL, A., MOLARO, G., ZILLI, L. & CLEMENTE, C. (1987). Ha-*ras*-1 restriction fragment length polymorphism and susceptibility to colon adenocarcinoma. *Br. J. Cancer*, **56**, 1.

FEINBERG, A.P. & VOGELSTEIN, B. (1983). A technique for radiolabelling DNA restriction endonuclease fragments to high specific activity. *Anal. Biochem.*, **132**, 6.

FIELD, J.K. & SPANDIDOS, D.A. (1987). Expression of oncogenes in human tumors with special reference to the head and neck region. *J. Oral Pathol.*, **16**, 97.

GUPTA, P.C., MEHTA, F.S., PINDBORG, J.J., AGHI, M.B., BHONSLE, R.B. & MURTI, P.R. (1988). An educational intervention study for tobacco chewing and smoking habits among Indian villagers. In *Smoking and Health, 1987*, Aoki, M., Hisamichi, S. & Tominaga, S. (eds) p. 623. Excerpta Medica: Amsterdam.

HEIGHWAY, Y., THATCHER, N., CERNY, T. & HASLETON, P.S. (1986). Genetic predisposition to human lung cancer. *Br. J. Cancer*, **53**, 453.

HONDA, K., ISHIZAKI, K., IKENAGA, M. & 4 others (1988). Increased frequency of specific alleles of the c-Ha-*ras* gene in Japanese cancer patients. *Hum. Genet.*, **79**, 297.

IKEDA, I., ISHIZAKA, Y., OCHIAI, M. & 5 others (1988). No correlation between L-*myc* restriction fragment length polymorphism and malignancy of human colorectal cancers. *Jpn. J. Cancer Res. (Gann)*, **79**, 674.

ISHIKAWA, J., MAEDA, S., KAMIDONO, S. & SUGIYAMA, T. (1988). Restriction fragment length polymorphism and activation of c-Ha-*ras* gene in urothelial cancer. *Anticancer Res.*, **8**, 915.

JUSSAWALLA, D.J. & DESHPANDE, V.A. (1971). Evaluation of cancer risk in tobacco chewers and smokers: an epidemiologic assessment. *Cancer*, **28**, 244.

KAKEHI, Y. & YOSHIDA, O. (1989). Restriction fragment length polymorphism of the L-*myc* gene and susceptibility to metastasis in renal cancer patients. *Int. J. Cancer*, **43**, 391.

KAWASHIMA, K., SHIKAMA, H., IMOTO, K. & 4 others (1988). Close correlation between restriction fragment length polymorphism of the L-*myc* gene and metastasis of human lung cancer to the lymph nodes and other organs. *Proc. Natl Acad. Sci. USA*, **85**, 2353.

KAWASHIMA, K., IMOTO, K., IZAWA, M. & 8 others (1987). Restriction fragment length polymorphism (RFLP) of L-*myc* is related to the progression of human colon and stomach cancers. *Proc. Jpn. Acad.*, **63**, 300.

KRONTIRIS, T.G., DIMARTINO, N.A., COLB, M. & PARKINSON, D.R. (1985). Unique allelic restriction fragments of the human Ha-*ras* locus in leucocyte and tumor DNAs of cancer patients. *Nature*, **313**, 369.

LIDERAU, R., ESCOT, C. THEILLET, C. & 4 others (1986). High frequency or rare alleles of the human c-Ha-*ras*-1 proto-oncogene in breast cancer patients. *J. Natl Cancer Inst.*, **77**, 697.

LIDERAU, R., MATHIEU-MAHUL, D., THEILLET, C. & 4 others (1985). Presence of an allelic EcoRI restriction fragment of the c-*mos* locus in leukocyte and tumor cell DNAs of breast cancer patients. *Proc. Natl Acad. Sci. USA*, **82**, 7068.

MANIATIS, T., FRITSCH, E.P. & SAMBROOK, J. (1982). *Molecular Cloning: a Laboratory Manual.* Cold Spring Harbor Laboratory: New York.

NAU, M.M., BROOKS, B.J., BATTEY, J. & 7 others (1985)). L-*myc*, a new myc related gene amplified and expressed in human small cell lung cancer. *Nature*, **318**, 69.

PINDBORG, J.J. (1984). Control of oral cancer in developing countries. *Bull. WHO*, **62**, 817.

SANGHAVI, L.D. (1981). Epidemiologic and intervention studies. Screening: cancer epidemiology: the Indian scene. *J. Cancer Res. Clin. Oncol.*, **9**, 1.

SARANATH, D., PANCHAL, R.G., NAIR, R. & 5 others (1989). Oncogene amplification in squamous cell carcinoma of the oral cavity. *Jpn. J. Cancer Res.*, **80**, 430.

SOUTHERN, E.M. (1975). Detection of specific sequences among DNA fragments separated by gel electrophoresis. *J. Mol. Biol.*, **98**, 503.

UNION INTERNATIONALE CONTRE LE CANCER TNM CLASSIFICATION OF MALIGNANT TUMOURS (1988). Ed. M.N. Harmer, Geneva.